# 1285. Identification of KPC Omega Loop Variant R163S Conferring Ceftazidime-Avibactam Resistance

**DOI:** 10.1093/ofid/ofab466.1477

**Published:** 2021-12-04

**Authors:** Alexander S Maris, Lili Tao, Paul Wada, Romney Humphries, Romney Humphries, Jonathan Schmitz

**Affiliations:** Vanderbilt University Medical Center, Nashville, Tennessee

## Abstract

**Background:**

We report on a 56 year-old male with prolonged COVID-19 pneumonia who initially improved with dexamethasone and intubation but quickly decompensated. Clinical and radiologic features were consistent with VAP. Tracheal aspirate cultures grew carbapenem-resistant Enterobacter cloacae; meropenem (MEM) MIC was >8 ug/ml (resistant) while ceftazidime-avibactam (CZA) MIC was 2/4 ug/ml (susceptible). Lateral flow antigen assay detected a KPC enzyme. The patient was treated with CZA with steady improvement in respiratory function over the next two weeks. He then experienced an episode of tachycardia, prompting repeat culture. At this point the patient had been extubated: sputum culture grew KPC+ E. cloacae that now showed CZA-resistance (MIC >8/4 ug/ml) and paradoxical decrease in MEM MIC (4 ug/ml); meropenem-vaborbactam (< 2/8 ug/ml) was susceptible.

**Methods:**

The pre- & post-CZA therapy E. cloacae isolates underwent whole genome sequencing using the Illumina 150bp paired end protocol; sequences were quality trimmed and compared.

**Results:**

A point mutation in the plasmid *bla*_*KPC3*_ gene was identified in the post-CZA therapy isolate, an R163S mutation in the omega loop of the enzyme. ompC and ompF porin genes were analyzed to rule-out decreased influx as a mechanism for CZA-resistance: the pre- and post-CZA isolates had identical porin sequences.

**Conclusion:**

This case highlights emerging mutations within KPC carbapenemases that lead to resistance to ‘last-line’ antimicrobials like CZA. The presumptive mechanism is increased KPC active site promiscuity due to increased omega loop flexibility, allowing increased ceftazidime binding and hydrolysis, and decreased avibactam binding and beta lactamase inhibition. Paradoxically, MEM susceptibility improves after such omega loop mutations, likely due to decreased active site binding affinity, a ‘seesaw’ effect between MEM and CZA. While authors have reported MEM MICs falling into the ‘susceptible’ category after an omega loop variant, these bacteria invariably develop secondary mutations leading to MEM treatment failure. Fortunately, given our patient’s improved respiratory status, the post-CZA E. cloacae isolate was felt to reflect colonization and the patient was discharged home without antimicrobial therapy.

**Disclosures:**

**Romney Humphries, PhD D(ABMM**), Accelerate Diagnostics (Individual(s) Involved: Self): Consultant, Shareholder; IHMA (Individual(s) Involved: Self): Consultant; Melinta (Individual(s) Involved: Self): Consultant; Momentum (Individual(s) Involved: Self): Grant/Research Support; Pattern (Individual(s) Involved: Self): Consultant; QPex (Individual(s) Involved: Self): Consultant; ThermoFisher (Individual(s) Involved: Self): Consultant; Torus (Individual(s) Involved: Self): Consultant

